# A cross-talk between integrin β4 and epidermal growth factor receptor induces gefitinib chemoresistance to gastric cancer

**DOI:** 10.1186/s12935-018-0548-5

**Published:** 2018-04-02

**Authors:** Jia Huafeng, Zhang Deqing, Ding Yong, Zhang Yulian, Hu Ailing

**Affiliations:** 1 Department of Gastroenterology, Hongze District People’s Hospital, Huai’an, 223100 Jiangsu China; 2 Department of Gastroenterology, The First Affiliated Hospital of Soochow University, Suzhou, Jiangsu China; 3 Department of General Surgery, Hongze District People’s Hospital, Huai’an, 223100 Jiangsu China; 4 Department of Oncology, Hongze District People’s Hospital, 102 Dongfeng Road, Hongze District, Huai’an, 223100 Jiangsu China

**Keywords:** Integrin β4, EGFR, Gastric cancer, Chemoresistance, Gefitinib

## Abstract

**Background:**

Gastric cancer presents a major health burden worldwide. Therefore, many molecular targeting agents have been evaluated for treatment of gastric cancer. Gefitinib has shown anticancer activity against gastric cancer which work through inhibiting epidermal growth factor receptor (EGFR). However, the effect of gefitinib is limited due to its resistance. Therefore, understanding the mechanisms of gefitinib resistance is desperately needed to formulate novel strategies against gastric cancer. Here, we analyzed resistance mechanism from the crosstalk between EGFR and integrin β4.

**Methods:**

Integrin β4-expression vector or siRNA were used to analyze the functional effects of integrin β4 on chemoresistance of gastric cancer cells to gefitinib. EGFR and integrin β4 expression, proliferation and apoptosis of gastric cancer cells were assayed by indirect immunofluorescence, western blot, MTT and flow cytometry respectively. EGFR and integrin β4 expression were also assayed on patient samples.

**Results:**

It was found that the integrin β4 expression was increased in gefitinib-resistant gastric cell line. The upregulated integrin β4 expression was identified to promote gefitinib resistance and proliferation, and inhibit apoptosis, while downregulation of integrin β4 was to inhibit gefitinib resistance and proliferation, and induce apoptosis. Moreover, the overexpression of integrin β4 in SGC7901 cells resulted in the down-regulation of p-EGFR protein levels while down-regulation of integrin β4, significantly resulted in overexpression of p-EGFR. The results of western blot from patients also showed there was obvious negative correlation between p-EGFR and integrin β4 in gastric cancer patients.

**Conclusion:**

Considering the above results, it is concluded that the interaction of EGFR and integrin β4 may change the sensitivity of gefitinib treatment.

## Background

Gastric Cancer arises from gastric mucosa which is lining of the stomach. Gastric cancer is usually associated with *Helicobacter pylori* infection. It is more common in developing countries. Gastric cancer is one of the world’s highest incidences of malignant tumors. It is one of the fourth most common cancers and the second leading cause of cancer-related deaths worldwide [1]. The occurrence and death rate of gastric cancer has shown a rapid increase over the last few years. Stomach cancer is easy to cure in early stages but difficult in advanced stages. The classic methods of treatment of gastric cancer include surgery, chemotherapy or both. However, these efforts are not so effective because gastric cancer in most patients is usually diagnosed at an advanced stage as early stages of gastric cancer cause few symptoms and lose the chance of radical resection. Therefore, further studies should be done regarding the complex biological behavior of gastric cancer to get better and effective treatment. Biological therapy and improved ways of using current methods are under observation as new treatment strategies.

The epidermal growth factor receptor (EGFR**)** is a transmembrane protein that allows the receptor to attach (bind) to other extracellular proteins, called ligands. It is a kind of glycoprotein receptor with tyrosine kinase activity on the surface of the cell membrane. The mutations in the EGFR lead to EGFR overexpression (known as upregulation) and its high expression can promote the proliferation, adhesion, invasion and metastasis of tumor cells [2] and is closely associated with non-small cell Lung cancer, breast cancer, gastric cancer, pancreatic cancer, colorectal cancer and other malignant tumors [3, 4].

In recent years, EGF receptor inhibitors such as gefitinib, erlotinib have obtained rapid clinical application because of high efficiency and low toxicity. The research related to EGF receptor inhibitors has become a focus in the field of malignant tumor. Gefitinib is one of the representative drugs which is a selective inhibitor of EGFR and typically acts as antiproliferative agent and induces cell apoptosis. Gefitinib is indicated for the treatment of patients suffering from cancer due to EGFR mutations but with clinical treatment the drug resistance is found in most of patients. Therefore, the resistance mechanism of the tumor invasion and metastasis is the main obstacle of cancer treatment.

Integrins are transmembrane receptors that facilitate cell-extracellular matrix (ECM) and cell–cell adhesion. These are important members of the family of adhesion molecules which play important roles in malignant tumor growth, invasion, and metastasis by mediating cell–cell and cell–matrix adhesion [5]. Integrins are prime regulators of adhesion, spreading, and migration of tumor. In tumor, epithelial cells express integrins which are present on surface of tumors. Integrins mediate adhesion of cancer cells with their surrounding and assist migration, proliferation and survival of tumor cells. The recent study shows that the β4 integrin related with α6 integrin contributes to the formation of hemidesmosomes in epithelial cells [6]. However, β4 integrin was primarily regarded as a tumor-associated protein that is present in metastatic cancer and contributes to the progression of tumor [7]. Assembles of hemidesmosome are blocked by EGF by phosphorylating the integrin β4 through MAPK signaling [8].

It is now believed that aberrant proliferation and inhibitory apoptosis are involved in drug resistance to cancer [9]. Therefore, in order to know the mechanism behind this, we tested interaction between EGFR and integrin β4 on the influence of proliferation and apoptosis of gastric cancer cell lines by blocking or promoting β4 synthesis with or without gefitinib. From this study, it was concluded that drug resistance is promoted through integrin β4 pathway in gastric cancer (adenocarcinoma).

## Materials and methods

### Cell lines

The gastric cancer cell line SGC7901 was cultured in RPMI-1640 medium and supplemented with 10% heat-inactivated fetal bovine serum (FBS), 100 IU penicillin/ml and 100 μg/ml streptomycin. The gefitinib-resistant variant SGC7901R was obtained from SGC7901 by exposing them to increasing concentrations of gefitinib (0.1, 1, 5 and 10 μM) (Sigma, MO, USA). Briefly, the cells were co-cultured with 0.1 μM of gefitinib for 24 h which was later on replaced by fresh medium. When living cells reached 80%, the monolayer cultures were passaged and cultured to logarithmic phase, then treated with 1 μM of gefitinib for 24 h. We repeated the same operations at the concentrations of 5 and 10 μM of gefitinib.

### Proliferation assay

SGC7901 and SGC7901R cells were seeded into a 96-well plate at a density of 1000/well and then incubated at 37 °C in 95% air and 5% CO_2_ respectively. After that, gefitinib was added to make the final concentrations of 0, 0.5, 1, 2 and 4 μM. After 48 h of incubation, the cell viability was then evaluated by MTT assay. 20 μl of MTT solution was added to 100 μl culture media which were further incubated for 4 h at 37 °C. Later on, 200 μl DMSO was added to each well after removing the culture medium and the plates were shaken for 10 min. The absorbance (OD) value was then measured at A490 nm. Three replicates of each sample were analyzed in each assay.

### Apoptosis assay

In order to assay apoptosis, SGC7901 or SGC7901R cells were treated with various chemicals and labeled with 4 μl of FITC-Annexin V and 8 μl of PI for 5-min in a dark place at room temperature. The specimens were then detected by using one flow cytometer for apoptosis assay. Normal living cells as well as early apoptotic cells resisted the staining by PI, but necrotic cells could not resist the staining [10].

### Integrin β4 siRNA or over-expressed vector

The full-length cDNA of integrin β4 was amplified from SGC7901/gefitinib cells and cloned into the *Xho*l I/*Bam*H I sites of the vector pcDNA3 in order to generate integrin β4 high expressed vector.

siRNA sequence targeting human integrin β4 was purchased from Santa Cruz biotechnology. The integrin β4 siRNA was transfected into gastric cancer cells with lipofectamine 2000. Corresponding negative-control siRNA was used as a negative control. After that, the total cellular extracts were prepared and the expressions of EGFR and integrin β4 were tested by western blot and real-time PCR.

### Real-time PCR

Real-time PCR monitored the amplification of a targeted DNA molecule during the PCR. RNA was removed from above samples by using Trizol so as to perform Real Time PCR. After RNA extraction, reverse transcription was performed by AMV reverse transcriptase. Real-time PCR was then carried out by using SYBR Green Real Time PCR Kit. The primers that were synthesized from the RNA are as follows: P-gp: 5′-ATGAGGGAGTATCTTCCTTACCTTCA-3, 3′-GCCCATCTCACCAACCAGTG-5; GAPDH: 5′-CCACTCCTCCACCTTTGAC-3′, 5′-ACCCTGTTGCTGTAGCCA-3. During this procedure, 1 µl of the reverse-transcriptase was added to a 2 µl PCR mixture for 40 cycles. Each cycle included 93 °C for 30 s, 60 °C for 60 s. Negative controls were used to compare the effect of a particular reaction. Negative controls consisted of an equal volume of water replaced for the volume of RNA in the RT reaction. The mRNA expression normalization data was achieved by comparing the copy numbers of target mRNAs with that of human GAPDH for each run [11].

### Western blot assays

Western blot assays are used to detect specific proteins in a tissue sample. In this study, western blot assays were used to monitor the expression of EGFR as well as integrin β4. The gastric cancer cell lines were cultured overnight to a confluence of 1 × 10^7^ cells/cm^2^ in RPMI-1640 medium containing 10% FBS before the experiment. Then the RPMI-1640 medium was removed and cells were arranged spherically as well as lyzed in lysis buffer. All the samples were centrifuged at 10,000 × rpm for 10 min. The protein was separated by sodium dodecyl sulfate–polyacrylamide gel electrophoresis. The Protein which was present on SDS-PAGE was placed to nitrocellulose and blocked with 3% bovine serum albumin in TBST. TBST is a mixture of tris-buffered saline (TBS) and Polysorbate 20. The protein was then added to rabbit anti-human antibodies. After that, it was rotated for 1 h at room temperature and washed with TBST three times. Finally an electrochemiluminescence kit including a goat anti-rabbit antibody detected the proteins on the nitrocellulose.

### Immunoprecipitation

Cells or clinic samples were lysed in RIPA buffer at 0 °C for 1 h, then centrifuged for 20 min at 15,000*g*. The supernatants were precleared with Sepharose 4B for 1 h and then incubated with monoclonal anti-EGFR antibody for 2 h followed by incubation with Sepharose 4B-conjugated secondary antibody. The Sepharose beads were washed three times, then boiled for 5 min in SDS-PAGE sample buffer. Samples were subjected to SDS-PAGE and western blotting by using mouse monoclonal anti-integrin β4 antibody.

### Clinic samples

Gastric cancer patients (16 men, 34 women) with 38 cases that were resistant to gefitinib were included in the study. The median age at surgery was 55 years old. The majority (70%) of patients had advanced TNM stage, and unexpected metastases were found in five cases (10%) during the surgery and postoperative pathology examination.

### Indirect immunofluorescence

After cells were grown to 80% confluence in 96-well round-bottom plates, anti-p-EGFR or anti-integrin β4 monoclonal antibody was added at 4 °C overnight followed by FITC-conjugated secondary antibody at 37 °C and 5% CO_2_ for 2 h, and then directly analyzed with fluorescence microscopy (Acasultm312,USA).

### Statistical analysis

The results were analyzed by ANOVA and Student’s *t* test. P value of less than 0.05 was considered statistically significant. Calculations were performed using SPSS software. Each experiment was repeated three times unless indicated otherwise.

## Results

### Integrin β4 associates with sensitivity of gefitinib of gastric cancer cell line

**Fig. 1:**
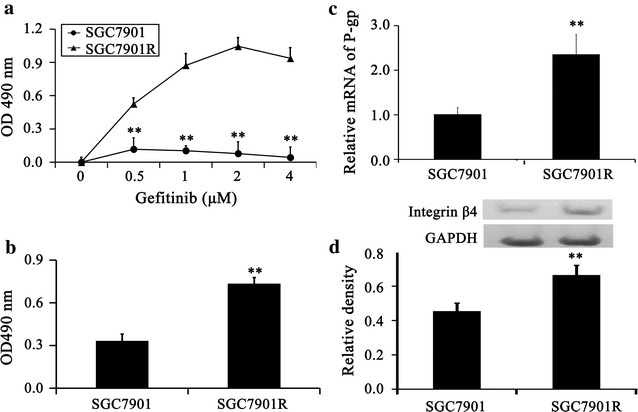
Integrin β4 associates with sensitivity of gefitinib of gastric cancer cell lines. **a** The sensitivity of the gastric cancer cell lines to gefitinib was assayed by MTT (n = 6). SGC7901R cells exhibited obvious resistance to gefitinib. **b** The proliferation of SGC7901R and SGC7901 was assayed by MTT (n = 6). The proliferation of SGC7901R was significantly up-regulated. **c** The resistant gene P-gp was assayed by real-time PCR. The expression of P-gp was increased significantly in SGC7901R cells. **d** The expression of integrin β4 was assayed by western blot (n = 4). The expression of integrin β4 was up-regulated in SGC7901R cells

In this study MTT assay was accomplished to find the sensitivity of the gastric cancer cell lines to gefitinib. The results of this experiment highlighted that the SGC7901R cells (IC50 = 2 μM) exhibited obvious resistance to gefitinib (Fig. 1a) compared with the SGC7901 cell line (IC50 = 50 nM). Moreover, the proliferation of SGC7901R cells was significantly higher than SGC7901 cells (Fig. 1b). To further verify the resistance of SGC7901R cells, P-glycoprotein (P-gp), which is recognized as the most widely observed mechanism in clinical multi-drug resistance of gastric cancer, was assayed. The results of real-time PCR showed that the expression level of P-gp increased significantly in SGC7901R cells (Fig. 1c). Western blot analysis was performed to decide the role of integrin β4 in the gefitinib-resistant gastric cancer cell lines. The results showed that integrin β4 expression was also higher in SGC7901R cells (Fig. 1d).

### Integrin β4 promotes proliferation and inhibits apoptosis

**Fig. 2:**
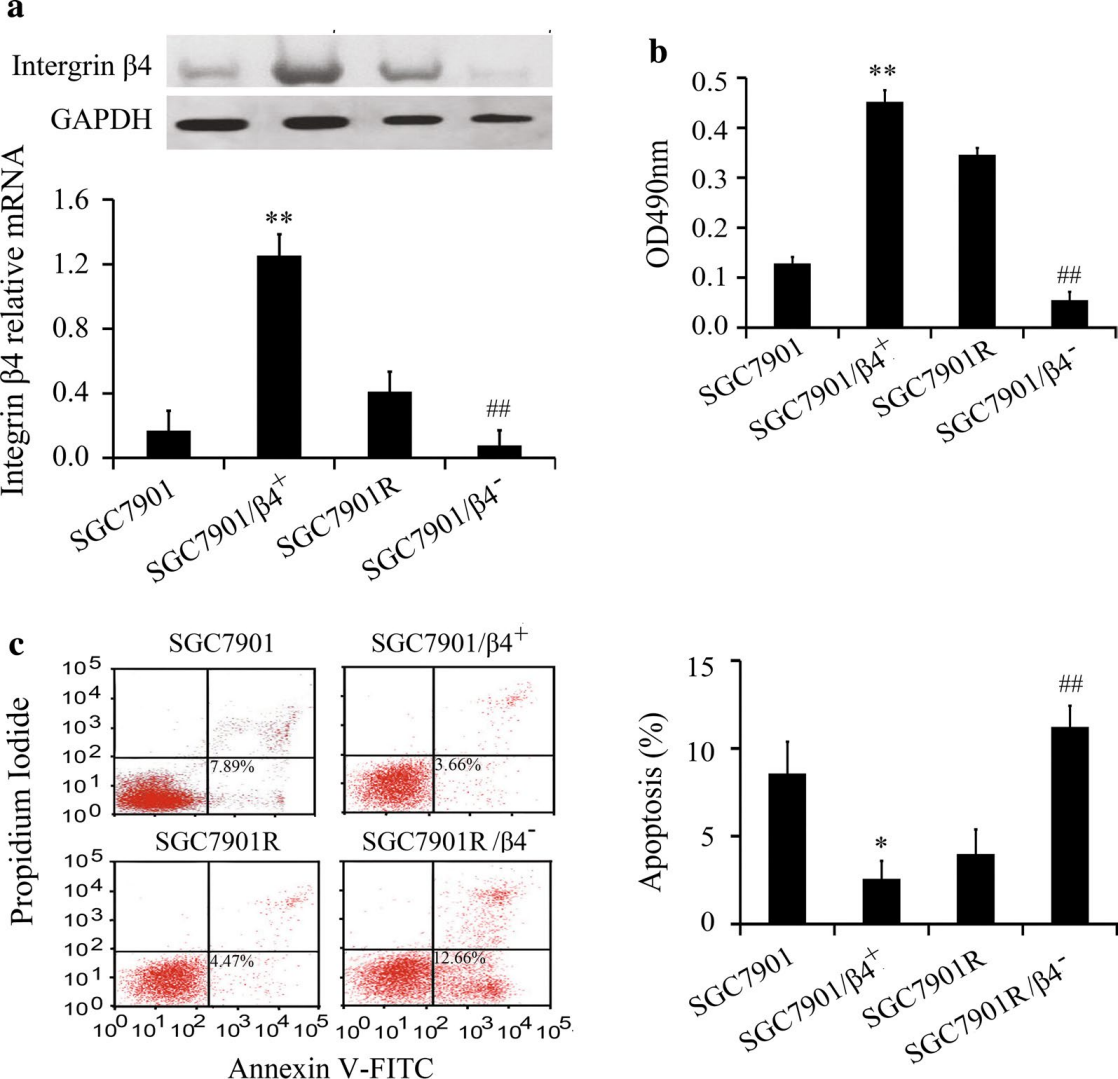
Integrin β4 promotes resistance of gastric cancer cell lines to gefitinib. **a** The expression of integrin β4 with integrin β4 siRNA or over-expressed vector was assayed by Western blot (n = 4) and real-time PCR (n = 4). **b** The proliferation of gastric cancer cell lines was assayed by MTT (n = 5). **c** Apoptosis rate was assayed by flow cytometry (n = 4). *P < 0.05 and **P < 0.01 vs. SCG7901. ^##^P < 0.01 vs. SGC7901R

To investigate the association between integrin β4 and gefitinib resistance, integrin β4 siRNA or overexpressed vector was transfected into gastric cancer cells. The expression of integrin β4 on cells was then examined by western blot and real-time PCR to verify whether the transfection was successful or not (Fig. 2a). After that, the proliferation of gastric cancer cell lines was evaluated by MTT Assay. The results indicated that the proliferation of integrin β4 siRNA cells was decreased while the proliferation of integrin β4-expression cells increased when compared with the corresponding controls (Fig. 2b). Moreover, integrin β4 siRNA promoted apoptosis while integrin β4-expression cells inhibited apoptosis which suggested that integrin β4 related with the sensitivity of gefitinib to gastric cancer cell lines (Fig. 2c).

### Integrin β4 modulates the expression of EGFR

**Fig. 3:**
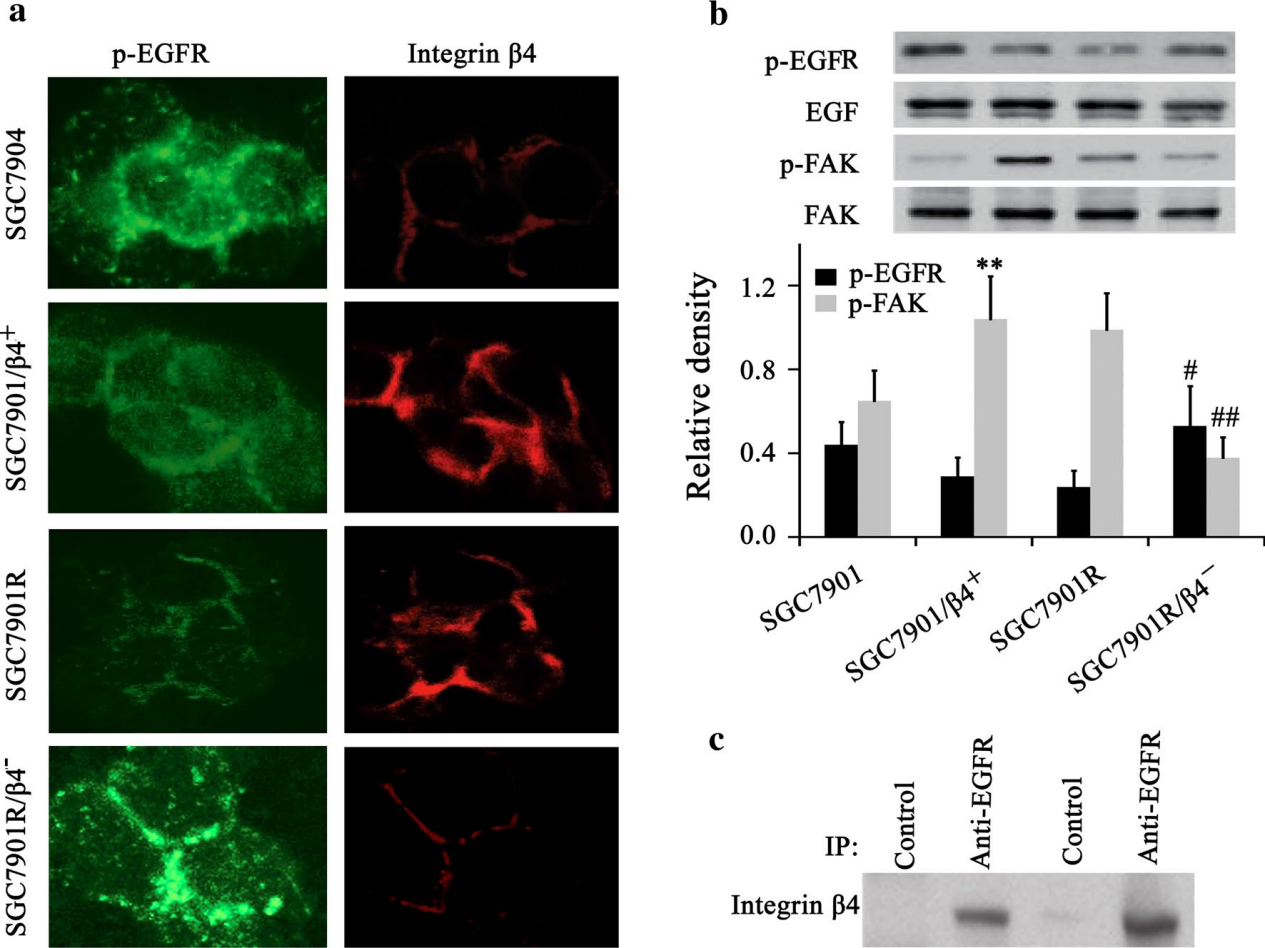
p-EGFR and intergrin β4 expression were assayed by IFA (**a**) and p-EGFR and p-FAK were assayed by western blot (**b**). Integrin β4 over expression induced rapid internalization of surface EGFR to the cytoplasm, while integrin β4 knockdown promoted the expression of EGFR. **c** The interaction between p-EGFR and intergrin β4 was assayed by immunocoprecipitation with anti-p-EGFR antibody. The first two samples were from SGC7901 cells and the second two samples were from clinic gefitinib-sensitive samples. *P < 0.05 vs. SCG7901. ^#^P < 0.05 and ^##^P < 0.01 vs. SGC7901R

The fluorescence microscopy was used to examine the pEGFR activation upon integrin β4 over expression or knockdown. The p-EGFR was detected with an anti-p-EGFR monoclonal antibody followed by FITC-conjugated secondary antibody. As shown in Fig. 3a, integrin β4 over expression induced rapid downregulation of p-EGFR while integrin β4 knockdown promoted the expression of p-EGFR. Western blot revealed that the bands corresponding to p-EGFR were increased in integrin β4 knockdown cell lines (Fig. 3b). The bands of p-FAK were increased in integrin β4 over expressed cell lines and diminished in integrin β4 knockdown cell lines (Fig. 3b). To further verify the interaction between EGFR and integrin β4, immunocoprecipitation by using anti-EGFR antibody was carried out on gastric cells and clinical samples. The results showed that integrin β4 can be detected in the precipitation by using western blot with anti-integrin β4 antibody (Fig. 3c), suggesting that the protein level of p-EGFR was regulated by integrin β4 signaling.

### Integrin β4 promotes gefitinib-resistance

**Fig. 4:**
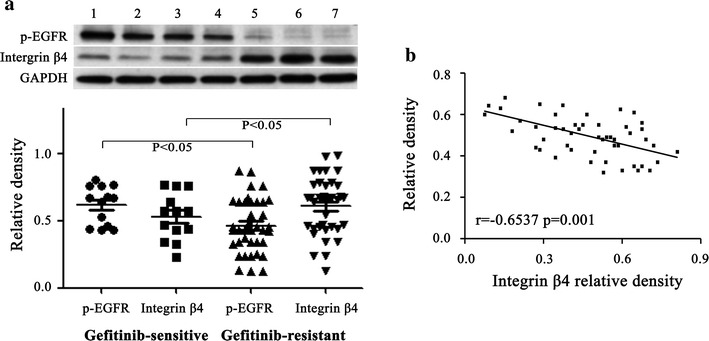
Integrin β4 was negatively correlated with p-EGFR activation (n = 50). **a** p-EGFR and integrin β4 protein expressions were determined by western blot. **b** Pearson correlation coefficients between p-EGFR and integrin β4. 1–4, gefitinib-sensitive gastric cancer samples; 5–7, gefitinib-resistant gastric cancer samples. *P < 0.05 vs. gefitinib-sensitive gastric cancer samples

The western blot was used to examine the expression level of integrin β4 in gefitinib-resistant gastric cancer patients. As shown in Fig. 4a, when compared with gefitinib-sensitive gastric cancer samples (n = 12), the expression of p-EGFR decreased and the expression of integrin β4 significantly upregulated in gefitinib-resistant samples (n = 38). Correlated analysis results showed p-EGFR were negatively associated with integrin β4 (r = − 0.6537, P < 0.01) (Fig. 4b).

It was recently announced that the drugs with certain mechanisms of action are targeted by molecules in tumors and are not so effective for common cancers. It was also announced that approaching a single target cannot inhibit the tumor progression in multisystem involving cancers such as colorectal cancer [12]. By analyzing these study results, it is shown that the failure or less effect of drugs may be caused by interaction of many signal pathways. In signal transduction pathways, one signal pathway is inhibited by molecular-targeted drugs, then, interaction from branches and shunting the signals from the trunk are being started thus making drugs less effective. In this study, it is showed that integrin β4 may be one of the branches to regulate the drug sensitivity in gastric cancer (adenocarcinoma), which is consistent with previous report that integrin β4 interacts with EGFR in a ligand independent manner and overexpression of integrin β4 induces drug resistance of hepatocellular carcinoma [13].

EGFR also known as ErbB1 or HER1, is a transmembrane protein having an extrinsic ligand-binding domain, a single transmembrane region with hydrophobic characteristics, and an intrinsic tyrosine kinase domain. Following ligand binding with EGFR, either homodimer or heterodimer can be formed thus causing auto-phosphorylation of tyrosine kinase and activation which initiates further signaling, ultimately resulting in DNA synthesis and cell proliferation. Integrin β4 is a heterodimeric cell adhesion receptor that mediates cells or cells to the extracellular matrix adhesion. EGFR and integrin β4 both have large cytoplasm domain containing several phosphorylation sites at serine and tyrosine residues, which is essential for their signaling coupling. FAK (Focal adhesion kinase) is a cytoplasmic non-receptor tyrosine kinase that modulates integrin-mediated signaling and various cell functions. According to Abdel-Ghany studies, in β4 integrin-related tumorigenesis and malignancy, β4 integrin facilitates the FAK-mediated signaling [14]. Elevated integrin β4 expression is associated with venous invasion and decreased overall survival in non-small cell lung cancer [15]. Moreover, Tai report an EGFR-dependent β4 integrin/FAK complex that contributes to malignancy of breast cancer, indicating the interaction between EGFR and β4 integrin [16]. In the present study, we verified there was direct interaction between p-EGFR and integrin β4. However, there was mutual regulation between p-EGFR and integrin β4 under resistance. We found that integrin β4 overexpression or gefitinib resistance both promoted the activation of FAK. Although integrin β4 knockdown inhibited the activation of p-FAK, it significantly promoted the activation of p-EGFR, indicating that there is a mutual modulation between integrin β4 and EGFR to induce the proliferation and growth of cancers. Furthermore, the negative correlation between these two molecules was also found in clinical samples indicating that a single molecular targeted drug to inhibit EGFR may induce resistance through upregulating the expression of integrin β4. Because of the interaction between EGFR and integrin β4, inhibition of a single target is not sufficient to block proliferation and growth of human adenocarcinoma cells of the stomach. However, what’s the molecular mechanism of integrin β4 and EGFR mutual regulation and what distinct signaling gets involved in the chemo-resistance need further study.

## Conclusion

In summary, our present study showes that there is a crosstalk between EGFR and integrin β4 pathways, which may induce drug resistance. Our report may give fresh ideas for treatment of gastric cancer with molecular-targeted therapy.
